# Understanding the relationship between couple dynamics and engagement with HIV care services: insights from a qualitative study in Eastern and Southern Africa

**DOI:** 10.1136/sextrans-2016-052976

**Published:** 2017-07-23

**Authors:** Joyce Wamoyi, Jenny Renju, Mosa Moshabela, Estelle McLean, Daniel Nyato, Doris Mbata, Oliver Bonnington, Janet Seeley, Kathryn Church, Basia Zaba, Alison Wringe

**Affiliations:** 1 National Institute for Medical Research, Mwanza, Tanzania; 2 London School of Hygiene and Tropical Medicine, London, UK; 3 Malawi Epidemiology and Intervention Research Unit, Karonga, Malawi; 4 Africa Health Research Institute, KwaZulu Natal, South Africa; 5 University of KwaZulu Natal, Durban, South Africa

**Keywords:** HIV, Couples, Relationships, Tanzania, Malawi, South Africa

## Abstract

**Objective:**

To explore the interplay between couple dynamics and the engagement of people living with HIV (PLHIV) with HIV care and treatment services in three health and demographic surveillance sites in Tanzania, Malawi and South Africa.

**Methods:**

A qualitative study was conducted involving 107 in-depth interviews with PLHIV with a range of HIV care and treatment histories, including current users of HIV clinics, and people not enrolled in HIV care. Interviews explored experiences of living with HIV and how and why they chose to engage or not with HIV services. Thematic analysis was conducted with the aid of NVivo 10.

**Results:**

We found an interplay between couple dynamics and HIV care and treatment-seeking behaviour in Tanzania, Malawi and South Africa. Being in a relationship impacted on the level and type of engagement with HIV services in multiple ways. In some instances, couples living with HIV supported each other which improved their engagement with care and strengthened their relationships. The desire to fulfil societal expectations and attract a new partner, or have a baby with a new partner, or to receive emotional or financial support, strengthened on-going engagement with HIV care and treatment. However, fear of blame, abandonment or abuse resulted in unwillingness to disclose and often led to disputes or discord between couples. There was little evidence of intracouple understanding of each other’s lived experiences with HIV, and we found that couples rarely interacted with the formal health system together.

**Conclusions:**

Couple dynamics influenced engagement with HIV testing, care and treatment for both partners through a myriad of pathways. Couple-friendly approaches to HIV care and treatment are needed that move beyond individualised care and which recognise partner roles in HIV care engagement.

## Background

Approximately two-thirds of people living with HIV (PLHIV) reside in sub-Saharan Africa,^1^ where unprotected heterosexual intercourse within the context of a relationship is a primary source of HIV infection.^1–3^ Relationship dynamics are influenced by social expectations that perpetuate gender differences resulting in differential utilisation of HIV services among men and women in many African settings.^4–7^ Furthermore, couple dynamics have been shown to have an important impact on adherence to antiretroviral therapy (ART).^4–6^

Over the past decade, strategies to improve ART uptake have focused on increasing treatment access through decentralisation of HIV services from secondary to primary facilities. The goal of such approaches has been to encourage more individual PLHIV to access ART free of charge close to where they live.^8 9^ However, although these policies aim to address individual and health system barriers to HIV service utilisation, they fail to acknowledge the social and family circumstances of most PLHIV.^10^

Little research has attempted to understand the particular circumstances of couples living together with HIV before, during or after the initiation of HIV treatment, beyond the dynamics of couple testing.^6 7 11 12^ The dearth of research evidence is mirrored in HIV care and treatment guidelines or programming tools, which rarely discuss the issues and challenges faced by couples. Indeed, aside from limited guidance on sexual behaviour and the promotion of condoms to prevent onward transmission, the notion of ‘couple’ has frequently been absent from HIV care and treatment programmes. Little is known about how couples engage in care and the bidirectional effect between being in a relationship and engaging in care.

### Theoretical perspectives

We consider that gender is socially constructed, and we use this theoretical lens to explore how people view themselves and their partners within the context of a heterosexual relationship. Women and men in sub-Saharan Africa are under pressure to fulfil the societal expectations that pertain to being a ‘respectable’ man or woman.^13 14^ For example, part of being ‘respectable’ and conforming to societal norms, is to marry and bear children.

We suggest that socially acceptable femininities and masculinities shape the decisions of men and women as they navigate HIV care and treatment and seek to initiate or maintain sexual relationships.^7 15^ As individuals receive an HIV diagnosis and initiate ART, they are challenged to manage the social expectations of what is ‘normal’ and ‘respectable’ behaviour for their gender within their relationships. These expectations may conflict with desires or demands to preserve their health, in particular when engaging with lifelong care and treatment that requires disclosing their HIV status to their partner.

**Figure 1:**
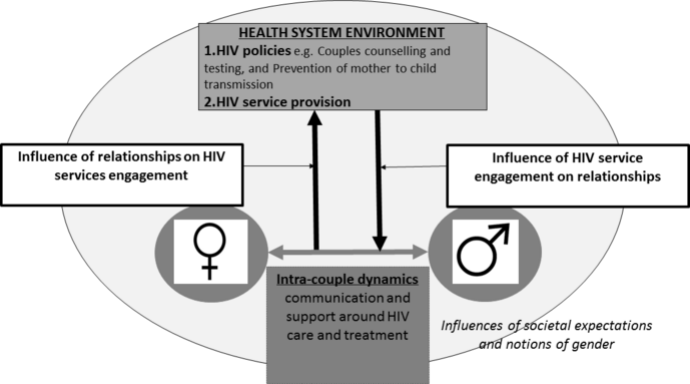
The interplay between couple dynamics and engagement with HIV care and treatment.

This paper explores the interplay between couple dynamics and the engagement of PLHIV with HIV care and treatment in three health and demographic surveillance sites (HDSS) in Kisesa (Tanzania), Karonga (Malawi) and uMkhanyakude (South Africa). We explore the complex interplay between PLHIV, their interactions within their relationships and their engagement in HIV care and treatment services (figure 1).

## Methods

Data are drawn from three of the HDSS (Kisesa, Karonga and uMkhanyakude) participating in the ‘Bottlenecks Study’ that aimed to investigate how differences in HIV policy and programme implementation influence the healthcare-seeking experiences of PLHIV through the continuum of HIV care in Eastern and Southern Africa. See additional information in the methods supplement included as part of the editorial found at http://dx.doi.org/10.1136/sextrans-2017-053172.

### Sampling and data collection

PLHIV were purposively sampled to represent different stages of HIV care and treatment engagement, ages and sex (table 1). Sampling frames were constructed from HDSS cohort datasets and clinic records. Additional methodological details concerning the Bottlenecks Study are provided elsewhere.

**Table 1:** Treatment history of participants by HDSS

Category	By health and demographic surveillance sites			Total
	Kisesa	Karonga	uMkhanyakude	
Diagnosed but not on antiretroviral therapy (ART)	13	9	10	32
Recently initiated on ART	7	7	10	24
On ART	10	20	7	37
Lost to follow-up	4	4	6	14
Total	34	40	33	107

Unlike the other two sites, the sample in Malawi included eight mutually disclosed couples. Couples were identified based on the initial interview with the first partner, after permission to approach their partner was granted. Face-to-face in-depth interviews with PLHIV took between 60 and 90 mins and were conducted in the local language, in private, in participants’ homes or in clinics by trained researchers. Topic guides focused on the experiences of PLHIV in their interactions with HIV services. Participants were prompted to recollect specific events related to their trajectory of care (for example diagnosis, ART initiation etc.) and asked to consider different influences on their experience of them.

### Data analysis

Data were transcribed, translated into English and coded at each site by the lead researchers, assisted by NVivo 10. The initial coding framework drew on the research objectives and wider literature. This was applied alongside a grounded theory approach to enable additional themes to be inductively identified.

Final analysis was conducted to identify emerging patterns, for example, exploring connections between ‘experiences of HIV care seeking’ and ‘relationship status’. Hypotheses were formulated, for example, the way individuals navigated HIV care depended on their desires to fulfil societal expectations of a respectable adult and elaborated by comparing data across the sites. Widespread views supporting emergent theories were examined alongside deviant cases.

## Results

A total of 107 PLHIV were interviewed, of whom 49% were women. Most (57%) were on ART, 30% had not yet started treatment and 13% had started ART but defaulted by the time of the interview. Table 1 shows this breakdown by HDSS.

### The influence of relationships on engagement with HIV care

#### Desire for couple relationships

The influence of relationships on engagement in HIV care was complex, sometimes promoting continued care seeking and other times undermining it. Social expectations around having children and being in a relationship were important drivers of care seeking among PLHIV. Since being ‘sick’ reduced one’s attractiveness to sexual partners, the desire to be healthy enough to attract a new partner or have a baby with a new partner, was often cited as driving on-going engagement with HIV care and treatment.

*My body condition was bad…you know, no man would dare touch me because of that condition…but now after taking these medicines [ART]…you can see I am now attractive…no one in this village can seduce me because they know my previous state…But when I go to the village centre they seduce me. [Female, On ART, Tanzania]*

Men more commonly hid their status in order to obtain a sexual or marital partner or sustain an on-going relationship.

*The fact that I loved her, I thought if I told her she will reject me…When I told her, she said if you would have told me before, we wouldn’t have married. [Male, on ART, Malawi]*

#### Power and gender within couple relationships

Asymmetrical power between partners promoted women’s engagement with care, with many women respecting their partner’s advice to test and seek care.

*My husband told me, ‘I am like this [HIV-infected] and you should also go to check your health’…So I just believed it because he told me to check. [Female, On ART, Tanzania]*

Likewise, some men drew on their powerful masculine role to ignore their partners’ requests to seek services or adhere to medication.

*He told me that before we met he got seriously sick. So I asked him to come with me but he refused. I think that’s how he does things. [Female, Pre-ART, South Africa]*

The linked couple analysis in Malawi illustrated that being in a supportive relationship in which the partners had disclosed their HIV status, improved both partners’ engagement in HIV care.

*I: What things help you remember taking your medicines every day?*

*R: What helps is that we are two so once you forget to take when we sleep we ask each other; ‘have you taken medicine?’ If the answer is ‘no’, you wake up, take in medicine and sleep. The same applies to everyone, so we remind each other. [Female, On ART, Malawi]*

However, this level of communication and support was rare, with many participants describing relationships characterised by mistrust and secrecy.

*She said I have also to get tested. I agreed that I will get tested, but I didn’t go for a test again because I already knew that l have HIV…Later I told her that I have also been found HIV positive and she was just quiet.[Male, pre-ART, Malawi]*

#### Infidelity

For some participants, suspicions of partner infidelity led to engagement in HIV care, notably by triggering a decision to undergo HIV testing. However, any benefit was lost when the drive to test was motivated by a need to assign blame about who introduced the virus into the relationship. Some participants feared potentially violent consequences of disclosure (voluntary or involuntary) driving them to hide their ART supplies, risking poor adherence or disengagement with care.

*I was scared of the man I was living with…I was taking [the drugs] at first and later threw them away because I was scared he would notice…He would have beaten me up or even killed me…I hid them in the maize flour…He still doesn’t know that I’m on treatment. [Female, on ART, Malawi]*

Similarly in Tanzania

*As soon as I came from the antenatal clinic I was afraid my husband would check in my bag so, I quickly removed them and threw them away…into the pit latrine because I was scared he would see them. [Female, On ART, Tanzania]*

### Influence of HIV service use on relationships

The same complexity was noted when exploring the influence of HIV service use on relationships, with disclosure emerging as a salient theme.

#### Disclosure in couple relationships

In some instances, disclosure of one’s HIV status and subsequent engagement in care encouraged closeness. Such disclosure was often driven by a need for support. Support given by partners included physical help when weak, collecting medication, reminders to take ART and accompanying partners to the clinic. This reportedly strengthened relationships for many participants. Some PLHIV actively sought out new partners of the same positive HIV status as a strategy to maintain a relationship whilst mutually and openly engaging in HIV care and treatment.

*…I desired to marry her after I found out about my status. I thought of looking for someone to stay with because sometimes I may get sick suddenly, so I needed someone to be closer to. [Male, on ART, Malawi]*

Prevention of mother-to-child transmission (PMTCT) and couple-testing policies were mentioned as drivers for improved couple communication, with some PLHIV reporting that they tried to test with their partner.

*After we got married…my wife……became pregnant and was diagnosed with HIV at antenatal care. When she came back during that time she disclosed this to me, so when they came and asked for consent, I freely wanted to know my status. This was what motivated me, but there was no sign of sickness. [Male, on ART, Malawi]*

However, diagnosis and initiation immediately onto treatment through antenatal care often further exacerbated challenges with partner disclosure, with many women not feeling adequately ready to accept or disclose their newly discovered status.

*I took the test and started taking the drugs…but until now…my husband doesn’t know. [Female, on ART, Tanzania]*

The analysis of the couple data from Malawi supported the findings from all the sites that most couples knew little about each other’s HIV status or treatment history. Most couples did not talk about HIV, either in terms of transmission risks, or HIV service use, perpetuating secrecy and mistrust within their relationships.

*My partner didn’t tell me that she was HIV positive, and she was on ART. But what was worrying me was that she was gaining weight, and I was losing weight. People told me that she is on ART, but she told me that she was taking tuberculosis treatment. [Male, on ART, South Africa]*

For women, apprehension and reluctance to disclose was heightened by the potential for physical violence, suspicions of promiscuity and loss of financial support. Guilt, shame and fear of negative consequences associated with HIV status disclosure were compounded by a fear of abandonment for most participants. There were several reports of relationships that broke up soon after an HIV diagnosis.

*I had to tell him: ‘I have to go [to my parents’] home because I’ve been suffering from intermittent fevers…I should go home to get treatment’…When I got home, I tested again…when those results came out, I was heartbroken…you know, I felt very lonely…I started [taking] the medicines and postponed going back there…I just stayed home and got rid of matters concerning a husband…and since then I haven't even been with a man. [Female, not on ART, Tanzania]*

*After I came back from the hospital he spoke these words, ‘you have killed me’ and those were his words and he said ‘from today I will not sleep in your house’. [Female, on ART, Malawi]*

For many, engagement or desire to engage in HIV care without partner/spousal support often resulted in relationship breakdown. Some couples reported that they decided to separate with their spouses after they felt sick and wanted to test for HIV, but the partner refused.

*I took the test and started taking the drugs…but until now…my husband doesn’t know. When I tell him to test, he becomes harsh…He doesn’t want to take the test at all…I always advise him every day and when I woke up today in the morning I told him to come along but, he doesn’t want…Now I have decided to leave him. [Female, On ART, Tanzania]*

## Discussion

We found a complex interplay between couple dynamics and HIV care and treatment-seeking behaviour in Tanzania, Malawi and South Africa. In some instances, couples living with HIV supported each other, which improved their engagement with care and strengthened their relationships. However, knowledge of each other’s HIV status could often lead to discord. There was little evidence of intracouple understanding of each other’s lived experiences with HIV. Our study highlighted that couples rarely interacted with the formal health system together, and this is a challenge for couple-focused interventions.^16^

Our findings suggest that gender norms enacted within relationships appear to challenge a woman’s ability to initiate a discussion about her status or advise their spouse to go for an HIV test. This is becoming an increasing problem as more women test through PMTCT programmes and discover their HIV status before their partners. Their male partners may resist participating in couple counselling if the relationship is insecure or to avoid relationship conflict.^6 17 18^ In a study in Uganda, Siu *et al* 19 reported that couple counselling threatened men’s masculinity because it meant disclosing their extramarital relations. Indeed, they found that the discussion of sexual risk and HIV with a spouse or other family members was believed to be inappropriate and threatening to one’s masculinity. Other authors have also described men’s tendency to avoid HIV services for fear of their wives’ negative reactions if they were found to have had extramarital relationships and risk being blamed for introducing HIV into the family.^7 20^

Insecurity and mistrust characterised many sexual relationships in our study and resulted in non-disclosure of one’s positive status. Many feared the consequences of their partner discovering their status and the fact they that they were on ART. Other studies have noted that couple disclosure can lead to the breakdown or dissolution of relationships,^10^ economic abandonment and intimate partner violence, particularly when women disclose to men.^21 22^ Men’s fear of blame for introducing the virus perpetuates an unwillingness to test^23^ and subsequently disclose their status to their partners. Other studies have shown that non-disclosure to close partners has been associated with late presentation to HIV care and non-adherence to ART.^7 24^

Fulfilling social norms surrounding adult unions could both promote and undermine PLHIV’s engagement in care. An HIV diagnosis seemed to change the sexual lives of many couples as some decided to abstain, reduce their number of partners, marry and/or terminate a relationship that they had been in for a long time. Marriage is an important social ideal;^13^ divorce or separation from one’s spouse can undermine reputations, especially for women, particularly if the separation was related to an HIV diagnosis. We found that the social values and roles associated with femininity create favourable conditions for women to engage with HIV services, while the reverse could be said for the values prescribed by masculinity on men.^13 25^ Interventions need to engage with couples in culturally appropriate ways.^16^ This could be achieved by addressing cultural norms relating to masculinity and femininity that might conflict with the health messages on HIV care among couples.

The study was strengthened by its positioning within HDSS in three countries. First, we were able to locate, recruit and interview PLHIV who were no longer in HIV care, although in small numbers. Second, in each setting, data saturation was achieved and the findings were similar across the different settings, lending support to the wider generalisability of our results. Third, in some instances, we were able to interview both partners in a couple. However, our study has a number of limitations. The study took place in rural settings, and couple dynamics and HIV care and treatment may be different in urban settings with more diverse sociodemographic profiles. Participants’ responses may have in some instances been influenced by social desirability bias. As with many qualitative studies, the small sample sizes (particularly when disaggregated by setting and treatment history) limited intercountry comparisons. Adopting a more ethnographic approach involving a larger sample of linked couples would allow more time and opportunities to unpick the complexities of relationships in the context of HIV infection in different settings.

## Conclusion

We found that couple dynamics and gender norms could positively, but more commonly negatively, influence engagement with HIV testing, care and treatment for both partners. Further consideration is required around how to engage partners in culturally appropriate ways to enhance HIV care and treatment uptake and adherence. This would involve approaches that address the complexities of PLHIV who are in relationships as they try to fit their health concerns and health-seeking behaviour within the acceptable cultural expectations in their communities, some of which may reflect contrasting moralities and dilemmas for PLHIV.

**Key messages**

- Many people living with HIV sought relationships to align their behaviour with social expectations, but this could have negative and positive influences on HIV care seeking.
- A fear of blame, abandonment or abuse resulted in an unwillingness to disclose and often led to disputes or discord between partners in relationships.
- Couple-friendly approaches to HIV care and treatment are needed that move beyond individualised care and which recognise partner roles in HIV care engagement.
